# Melanin Correction Is Essential for Quantitative Autofluorescence-Based Measurement of Macular Pigment

**DOI:** 10.3390/diagnostics16121751

**Published:** 2026-06-06

**Authors:** Mohsen Sharifzadeh

**Affiliations:** Department of Electrical and Computer Engineering, Brigham Young University, Provo, UT 84602, USA; sharifzadeh.mohsen@gmail.com or msh1344@byu.edu

**Keywords:** age-related macular degeneration, retinal biomarkers, diagnostic imaging, macular pigment optical density, autofluorescence imaging, melanin correction, retinal pigment epithelium, supplementation studies

## Abstract

Macular pigment optical density (MPOD) is widely measured by dual-wavelength autofluorescence imaging (AFI) because the method is noninvasive, image-based, and clinically practical. AFI-derived MPOD and macular pigment optical volume (MPOV) are increasingly used as retinal biomarkers in clinical research, supplementation studies, disease-risk interpretation, and population-based comparisons. However, conventional dual-wavelength AFI assumes that posterior absorbers other than macular pigment (MP), particularly melanin, are negligible or sufficiently stable not to bias the measurement. This assumption may limit the diagnostic and biomarker reliability of AFI-derived MP metrics. This manuscript presents a focused biological, optical, and mathematical analysis of AFI-based MP quantification. The foundational AFI literature, the melanin imaging literature, retinal pigment epithelium (RPE) pigment biology, and related optical modeling concepts were examined to evaluate where melanin enters the AFI signal pathway, how it may confound MPOD and MPOV, and how a melanin-sensitive baseline could improve quantitative specificity within the AFI domain. Conventional dual-wavelength AFI estimates MP indirectly from attenuation of RPE lipofuscin autofluorescence under excitation wavelengths that differ in MP absorption. Because melanin is located in the RPE and choroid, varies with retinal location, age, and pigmentation, and can influence the same excitation and detection pathway, unmeasured melanin can become embedded in the apparent MPOD signal. Under these conditions, reported MPOD and derived MPOV are better understood as model-dependent estimates whose quantitative specificity may vary across subjects, retinal locations, devices, and studies. This has direct implications for diagnostic interpretation, normative databases, cross-subject comparison, supplementation-response studies, and biomarker-based retinal assessment. Melanin correction is not a minor refinement of AFI-based MP quantification. It is likely necessary when AFI-derived MP metrics are intended to be interpreted as quantitatively specific retinal biomarkers rather than conditionally approximate optical estimates. A melanin-corrected AFI framework, based on introducing a melanin-sensitive baseline wavelength outside the principal MP absorption range, offers a path toward more reliable MPOD and MPOV interpretation in clinical, diagnostic, and supplementation-related studies.

## 1. Introduction

Macular pigment (MP), composed primarily of lutein, zeaxanthin, and meso-zeaxanthin, is concentrated in the central retina and has long been studied because of its optical and biological relevance. Its short-wavelength absorption, antioxidant role, and possible relationship to retinal health have made quantitative MP measurement important in both research and clinical settings, particularly in studies of aging, supplementation, and macular disease. As interest in personalized retinal risk assessment and intervention has grown, so has the need for methods that provide reproducible and biologically valid estimates of macular pigment optical density (MPOD) [1,2]. This need is particularly important in supplementation-related clinical studies and retinal biomarker research, where AFI-derived MP metrics are often used to compare baseline status, treatment-associated changes, disease-risk profiles, or population-level differences across subjects.

Among the available approaches, autofluorescence-based methods gained major importance because they provide a noninvasive, single-pass optical strategy for estimating MPOD by exploiting lipofuscin autofluorescence from the retinal pigment epithelium (RPE). In the classical framework, lipofuscin fluorescence is excited at two wavelengths, one more strongly absorbed by MP and the other minimally absorbed by MP, while emission is detected outside the principal MP absorption range. This approach has been highly influential because it can provide either central MPOD estimates or spatial maps of MP distribution, and it has supported a substantial body of the clinical and translational literature [1,2].

However, the apparent specificity of conventional autofluorescence-based MP measurement depends on assumptions that are biologically and optically fragile. In particular, the classical formulation assumes that foveal–perifoveal differences in other absorbers posterior to MP, including RPE melanin, are negligible or sufficiently stable not to bias the result. Yet melanin is a major ocular chromophore located in the RPE and choroid, contributes substantially to retinal optical behavior, changes with age and disease, and remains difficult to quantify directly in vivo with standard clinical instrumentation. More broadly, melanin-related contrast has motivated the development of multiple dedicated retinal imaging approaches, which itself reflects that melanin is neither trivial nor optically silent in fundus-based measurements [1,3].

This issue matters because an autofluorescence-derived MPOD value is often interpreted as though it were a direct quantitative measure of macular carotenoid absorption alone. If melanin is not independently estimated, that interpretation may be incomplete. The measured signal can instead reflect a mixed optical system in which MP, lipofuscin, and melanin all shape excitation and detected emission to varying degrees, depending on wavelength selection, retinal location, pigmentation, aging, and device design. Under such conditions, absolute MPOD values may become population-dependent, wavelength-dependent, and not fully comparable across studies or platforms. The problem is therefore not that prior autofluorescence work lacks value, but that the field has generally underappreciated the extent to which melanin can confound truly quantitative MP measurement [2].

The purpose of this manuscript is to examine that neglected confound in a systematic way. We review the biological and optical basis for melanin interference in autofluorescence-based MP measurement, analyze why conventional dual-wavelength formulations are not fully sufficient for quantitative specificity when melanin is unmeasured, and outline a correction framework that remains within the autofluorescence imaging domain. The central argument is not that prior methods should be discarded, but that biologically valid quantitative autofluorescence-based measurement of MP requires explicit consideration of melanin. Only then can MPOD estimates be interpreted with greater confidence across subjects, devices, and clinical studies. This issue is especially relevant to supplementation, diagnostic imaging, and biomarker-oriented retinal studies, where cross-sectional interpretation of AFI-derived MPOD or macular pigment optical volume (MPOV) may be misleading if posterior melanin is not independently accounted for. In such settings, uncorrected AFI-derived MP metrics may affect not only optical quantification, but also the interpretation of retinal biomarker status, normative comparisons, and clinically relevant group differences.

## 2. Biological and Optical Basis of the Problem

Any quantitative discussion of autofluorescence-based MP measurement must begin with the fact that the detected signal does not arise from a single isolated chromophore. Rather, it emerges from a layered retinal system in which MP, lipofuscin, and melanin occupy different anatomical compartments, have different spectral behaviors, and influence the propagation of excitation and emitted light in different ways. Classical autofluorescence methods took advantage of this layered structure in an elegant and clinically useful manner, but the same structure also creates the central limitation addressed in this manuscript: the measured signal cannot be assumed to reflect MP alone when other posterior absorbers are present and spatially nonuniform [1,2].

### 2.1. Macular Pigment Location and Spectral Behavior

Macular pigment is a short-wavelength absorbing retinal pigment concentrated in the central macula, with greatest density at the fovea. In the classical optical description, it is located predominantly in the cone axons and related central retinal layers, where it acts as a pre-receptoral filter for blue light. Its absorption spectrum extends broadly across the blue-green range, approximately 400–540 or 550 nm, with a peak near 455–460 nm. This spectral profile is the basis for optical MPOD measurement, because excitation wavelengths within this range are attenuated by MP before reaching deeper retinal structures [1,2].

This anatomical position is crucial. Because MP lies anterior to the retinal pigment epithelium, it attenuates the excitation light used to stimulate fundus autofluorescence from deeper fluorophores, particularly lipofuscin in the RPE. Conventional autofluorescence-based measurement therefore infers MPOD from the reduction in detected autofluorescence under an excitation wavelength strongly absorbed by MP relative to a second wavelength minimally absorbed by MP. In this framework, MP is not measured directly; it is estimated from its effect on light delivery to posterior tissue. That logic is powerful, but it also means that any additional absorber or wavelength-dependent optical difference posterior to MP can influence the inferred result [1,2].

The spatial distribution of MP further complicates the problem. MP is not uniform across the posterior pole but rises steeply toward the foveal center and declines with eccentricity, often within the central few degrees. Autofluorescence imaging exploits this gradient to generate topographic MP maps, and the central darkening seen under short-wavelength excitation is one of the most recognizable signatures of MP. However, that darkening is interpreted against a background signal produced in deeper tissue, not in isolation. The quantitative specificity of the resulting MPOD map therefore depends not only on the absorption spectrum of MP itself, but also on the stability and separability of the deeper contributors that shape the baseline autofluorescence signal [2].

### 2.2. Lipofuscin as the Autofluorescence Source in Conventional MP Imaging

Conventional autofluorescence-based measurement of MP relies on a deeper retinal fluorophore, not on intrinsic fluorescence from macular carotenoids themselves. In practice, the dominant signal source is lipofuscin within the retinal pigment epithelium, whose broad fundus autofluorescence can be excited over much of the blue-green range and detected at longer wavelengths where MP absorption is minimal. This is the central reason autofluorescence imaging became attractive for MP measurement: it converts the RPE into an internal light source and allows MP to be inferred indirectly from its attenuation of excitation reaching that deeper layer [2,4].

This indirect logic is elegant, but it is also the source of the present problem. Because the measured signal originates in the RPE rather than in the MP layer itself, the final MPOD estimate depends on the optical path between the excitation source, the overlying retinal layers, the lipofuscin-containing RPE, and the detected emission path back to the camera or detector. In other words, autofluorescence-based MP imaging is fundamentally a layered transmission problem, not a direct molecular readout of macular carotenoids. That distinction is critical, because any chromophore posterior to MP that alters excitation delivery or emission recovery can influence the inferred MP value [2,4].

Lipofuscin itself is not optically trivial. It accumulates with age in RPE cells, is spatially nonuniform across the fundus, and shows different topographic behavior from melanin, including a characteristic increase toward the posterior pole with a relative dip at the fovea. Earlier histologic work also showed an inverse relationship between RPE lipofuscin and RPE melanin, which is highly relevant here because it means that the autofluorescence source and one of its main confounders are not distributed independently. Thus, even before one reaches the mathematical problem of MP estimation, the biology of the RPE already suggests that a simple two-state interpretation of the signal is incomplete [5].

A further point is important for the tone of this manuscript. The dependence of MP measurement on lipofuscin autofluorescence does not make the conventional method weak; in fact, it is precisely what made the method clinically practical and highly influential. The limitation is different: once MP is estimated from modulation of a deeper fluorescence source, quantitative accuracy depends on whether the other optical determinants of that source have been sufficiently modeled. That is the point at which melanin becomes unavoidable.

### 2.3. RPE and Choroidal Melanin: Anatomy, Distribution, and Age-Related Relevance

Melanin is not a minor background absorber in the posterior eye. It is a major ocular pigment located primarily in the retinal pigment epithelium and choroid, where it contributes to light absorption, reduction in stray light, and protection against photo-oxidative stress. In the RPE, melanin is contained within melanosomes that are formed early in life and are generally regarded as far less regenerative than many other cellular constituents. This makes melanin biologically important not only because of its optical properties, but also because age-related or disease-related changes in its amount, localization, or functional state may persist over long time scales [3,5].

Histologic and optical studies have shown that melanin is distributed nonuniformly across the posterior pole and cannot be treated as spatially constant. In classic human donor-eye measurements, RPE melanin concentration decreased from the periphery toward the posterior pole but showed an increase in the macular region, whereas choroidal melanin increased toward the posterior pole and was consistently greater overall than RPE melanin on a per-area basis. The same study also reported that choroidal melanin was significantly greater in eyes from Black subjects than in eyes from White subjects, whereas RPE melanin was more similar between the two groups. These findings are directly relevant to autofluorescence-based MP measurement because they show that the optical environment posterior to MP varies both topographically and across pigmentation groups [5]. This spatial nonuniformity is especially relevant because AFI-based studies often report eccentricity-dependent MPOD or derive MPOV from spatial MP maps, both of which may be affected if posterior melanin varies across the macula.

Melanin also does not vary independently of other RPE pigments. Earlier work demonstrated an inverse relationship between RPE melanin and lipofuscin, both topographically and across measurement sites, implying that the principal autofluorescence source used in conventional MP imaging and one of its major confounders are biologically linked rather than separable by assumption alone. In addition, aging changes this balance. Lipofuscin generally increases with age, whereas both RPE and choroidal melanin show trends toward reduction or redistribution, and melanin-related autofluorescence behavior may also change with oxidation and compartmental shifts. Thus, the posterior optical baseline underlying autofluorescence imaging is not fixed across the lifespan [5,6].

A second important point is methodological. The fact that multiple dedicated technologies have been developed to visualize or estimate ocular melanin in vivo, including fundus reflectometry, near-infrared autofluorescence, photoacoustic approaches, and melanin-sensitive OCT variants, already indicates that melanin is a distinct and meaningful imaging target rather than a negligible residual term. At the same time, no single standard clinical technique has fully resolved the challenge of quantitative melanin measurement across eyes. This combination of biological importance, optical influence, and limited direct quantifiability in vivo is exactly what makes melanin a critical confound in quantitative autofluorescence-based measurement of MP [3].

### 2.4. Why These Chromophores Cannot Be Treated as Optically Independent in Practice

The central optical difficulty is that autofluorescence-based MP measurement is not generated by a single absorber acting on a single fluorophore in an otherwise neutral background. Rather, the method operates in a layered posterior segment in which MP attenuates excitation light before it reaches the RPE, lipofuscin provides the dominant detected autofluorescence signal, and melanin within the RPE and choroid modifies the same optical pathway through wavelength-dependent absorption and related pigment-linked effects. Delori’s classical framework already recognized that the RPE contains both lipofuscin and melanin and explicitly noted that RPE melanin can cause overestimation of MP optical density [1,2].

This means that the usual conceptual separation of chromophore roles is only approximate. Macular pigment is treated as the modulator of excitation, lipofuscin as the source of emission, and melanin as a secondary background influence. In practice, however, those roles overlap. The excitation wavelengths used for conventional AFI are chosen because they probe the difference between stronger and weaker MP absorption, but those same wavelengths also interact with melanin and with the local optical environment of the RPE. Because the measurement is based on differences between foveal and perifoveal signals, any topographic variation in melanin or in the lipofuscin-melanin balance can enter the MPOD estimate unless it is explicitly modeled [2,5]. This concern extends beyond a single central MPOD value to eccentricity-based MP profiles and integrated spatial measures such as MPOV.

The problem is not merely theoretical. Histologic work has shown that lipofuscin and melanin are related inversely across the posterior pole, while melanin itself varies by region, age, and pigmentation group. At the same time, melanin is sufficiently optically distinct that separate imaging approaches have been developed specifically to visualize or estimate it in vivo, including near-infrared autofluorescence and other melanin-sensitive techniques. These observations argue against treating melanin as a negligible constant term in quantitative AFI. If a pigment requires dedicated imaging methods to be visualized and is known to vary biologically across eyes, it should not be assumed away in another quantitative optical method that uses the same posterior tissues [5].

From a measurement standpoint, the consequence is that conventional dual-wavelength AFI is an underconstrained system when melanin is not independently estimated. The measured autofluorescence ratio can reflect not only MP attenuation but also melanin-linked attenuation differences embedded in the same signal path. A proposed solution within the autofluorescence domain is to incorporate a melanin-sensitive baseline, allowing melanin absorption to be estimated and then translated to the MP-sensitive wavelengths before calculating corrected MPOD. This general strategy has been described in a published patent framework by the present author. Whether implemented with two or more excitation wavelengths, the key principle is the same. A quantitative MP measurement cannot be considered optically specific if one of the major posterior absorbers remains unmeasured [7].

## 3. What Conventional Dual-Wavelength AFI Actually Measures

The widespread clinical appeal of dual-wavelength autofluorescence imaging lies in its apparent simplicity. A short excitation wavelength that is strongly absorbed by MP is compared with a second excitation wavelength that is minimally absorbed by MP, and the resulting autofluorescence difference is used to estimate MPOD. This framework has been highly influential because it enables topographic visualization of MP and has shown practical utility and reproducibility in both research and clinical settings [2,8]. However, the quantity produced by this method is not a direct molecular measurement of macular carotenoids. It is an inferred optical estimate derived from the behavior of a layered posterior segment signal. For that reason, it is essential to define clearly what the method measures, what assumptions it requires, and where those assumptions may fail.

### 3.1. Core Principle of Ordinary AFI MPOD Estimation

In conventional dual-wavelength AFI, the measured signal is based on lipofuscin autofluorescence arising from the retinal pigment epithelium after excitation at two wavelengths with different sensitivity to MP absorption. One wavelength is selected within a range where MP absorbs strongly, while the second is chosen where macular pigment absorption is much weaker. Because MP lies anterior to the RPE, it attenuates the shorter excitation more strongly before that light reaches the lipofuscin-containing layer. The resulting reduction in autofluorescence is then interpreted as evidence of greater MP density in the overlying retina.

In its classical form, the method compares foveal and perifoveal autofluorescence under the two excitation conditions. The perifovea is typically used as a reference region under the assumption that MP is low enough there to approximate a baseline, whereas the fovea shows stronger attenuation at the macular-pigment-sensitive wavelength. By normalizing the difference between these signals, the method yields an estimate of MPOD or a spatial map of MP distribution. The clinical attractiveness of this approach is obvious. It is noninvasive, image-based, and capable of producing intuitive maps of central pigment distribution.

However, the quantity being extracted is not simply MP absorption. It is more accurately the relative change in detected RPE autofluorescence under two excitation conditions after the light has passed through a complex optical system. The final value therefore depends on several linked factors, including the excitation spectrum of the instrument, the spectral absorption of MP, the spatial distribution of the autofluorescence source, the optical properties of tissues posterior to MP, and the assumptions used in selecting the reference region and normalization procedure.

That distinction becomes especially important when the result is interpreted quantitatively. If all other relevant contributors were constant or negligible, the inferred value could closely track true MPOD. But if another posterior absorber contributes to wavelength-dependent attenuation within the same pathway, then the measured dual-wavelength ratio no longer represents MP alone. Under those conditions, ordinary AFI still provides a useful apparent MPOD estimate, but its specificity as a quantitative MP measurement becomes conditional rather than absolute.

### 3.2. Explicit Assumptions in the Classical Framework

The classical dual-wavelength AFI formulation is elegant because it reduces the problem to a limited number of optical terms. However, that simplification depends on several assumptions that must hold if the resulting MPOD estimate is to remain specific to MP. In Delori’s formulation, the fluorescence ratio is interpreted as MP-dependent only if the foveal and perifoveal fluorescence efficiencies remain proportional over the excitation range employed, and if foveal-perifoveal differences in absorption by other pigments are negligible. Importantly, those other pigments are identified explicitly in the original framework and include retinal blood, visual pigments, and RPE melanin [1].

A second assumption is that the underlying autofluorescence source behaves sufficiently similarly at the fovea and the reference region. In practical terms, this means that the lipofuscin-related fluorescence efficiency should not differ in a way that introduces wavelength-dependent bias unrelated to MP itself. Delori noted this point directly by stating that the fluorophore at the fovea is assumed to have the same composition as that at the perifovea, meaning a constant excitation spectrum shape across locations. He also stated that little was known about whether lipofuscin composition changes with retinal location [1].

A third assumption concerns the reference site. The perifovea is used operationally as a low-MP baseline, but this choice also assumes that the reference eccentricity is otherwise suitable for normalization and that non-MP contributors remain sufficiently stable between the measuring and reference sites. This point appears again in the later clinical literature. The South Indian population study noted that the method relies on univariance, meaning that MP is assumed to be the only difference between the measuring and reference sites, and suggested that this could be a general failing of the technique [8].

Importantly, good repeatability does not eliminate these assumptions. Clinical studies using Spectralis-based dual-wavelength AFI have shown high reproducibility, which is valuable and supports practical utility [9]. However, reproducibility is not the same as full quantitative specificity. As the reproducibility study itself noted, the accuracy of dual-wavelength autofluorescence measurement has not been validated against an in vivo gold standard, and more broadly there is no clear in vivo gold standard for MPOD measurement [4,9,10]. Thus, a method may reproduce its own output consistently while still carrying a systematic bias from an unmeasured confounder.

Taken together, these assumptions define the precise point at which the classical framework becomes vulnerable. If non-MP absorbers are negligible and posterior fluorescence behavior is sufficiently stable, the method can perform as intended. But if melanin contributes non-negligible, spatially variable, and wavelength-dependent attenuation within the same optical pathway, then the dual-wavelength AFI estimate is no longer determined by MP alone. The issue, therefore, is not whether the classical framework is useful. It clearly is. The issue is that its quantitative interpretation becomes conditional on assumptions that are not guaranteed across eyes, ages, pigmentation levels, and devices [2,8].

### 3.3. Where Melanin Enters the Excitation and Detection Pathway

Melanin enters the conventional AFI measurement pathway at the same anatomical level from which the autofluorescence signal is generated. In the classical model, detected fundus autofluorescence arises predominantly from lipofuscin in the RPE, while MP attenuates the excitation light before it reaches that layer. However, melanin is intermixed with lipofuscin within the RPE and is also present posteriorly in the choroid, so it can influence the same optical path by attenuating excitation light and, depending on the geometry and wavelength range, the returning signal as well [1,2,5]. In other words, melanin is not external to the AFI measurement. It is embedded within the posterior segment structure that generates and shapes the measured signal.

This point was addressed directly by Delori and colleagues. In the reflectometry and autofluorescence spectrometry study, they noted that RPE lipofuscin is intermixed with melanin in the RPE cell, causing partial attenuation of both excitation and emission from lipofuscin, and they further showed that AF-derived MP density exhibited a significant positive correlation with independently estimated melanin density difference. They then derived an explicit correction relation for the AF method to account for melanin, concluding that uncorrected AF-based MP estimates are affected by RPE melanin [1]. Thus, the possibility that melanin contaminates quantitative AFI is not a speculative criticism of the classical method. It is already present within the method’s own optical logic and acknowledged in the foundational literature.

The importance of this pathway dependence becomes even clearer when one considers wavelength choice. Conventional MP imaging commonly uses blue-green excitation, such as 488 nm and 514 nm, because these wavelengths interact strongly enough with MP to produce measurable differential attenuation. But melanin also has substantial absorption in this spectral region, with absorption decreasing toward longer wavelengths. As a result, differences in melanin amount or distribution between the fovea and reference region can become embedded in the same dual-wavelength contrast used to estimate MPOD. A longer excitation wavelength above the main MP absorption range can therefore provide information that is much less affected by MP while still retaining melanin-related attenuation, which is precisely why a melanin-sensitive baseline is conceptually valuable within an AFI framework [1,7].

A related point is that melanin is not merely an absorber inferred indirectly from reflectance theory. Ocular melanin can itself be visualized by dedicated imaging approaches, especially near-infrared autofluorescence, where the signal arises predominantly from melanin or melanin-associated compounds in the RPE and, to a varying degree, the choroid [3,6]. The existence of these melanin-sensitive imaging modalities reinforces a simple methodological lesson. If melanin is sufficiently optically active to produce its own measurable retinal imaging contrast, it cannot reasonably be assumed to be irrelevant in another quantitative fundus imaging method that probes the same tissues with shorter excitation wavelengths.

**Figure 1:**
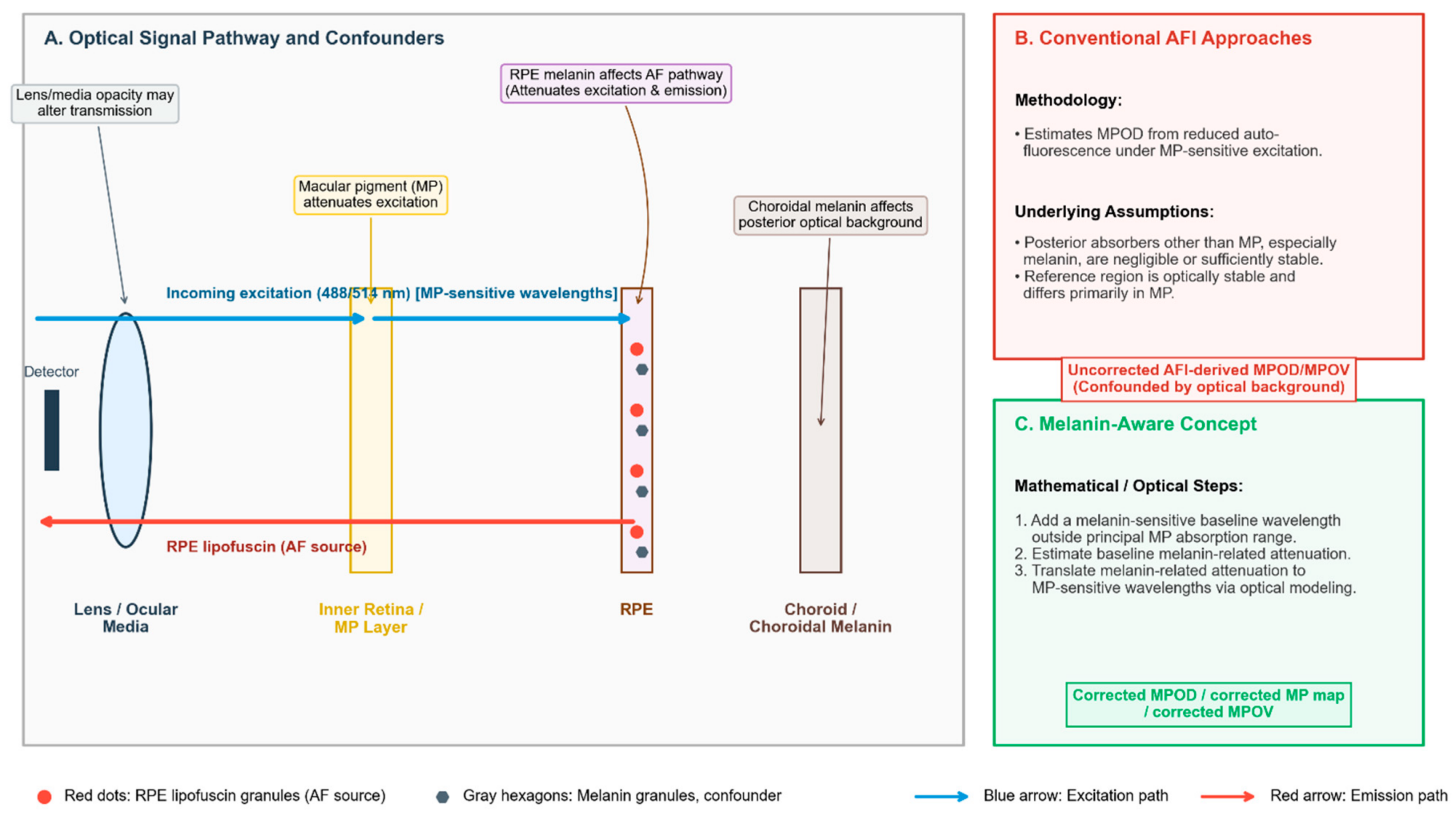
Optical pathway of conventional dual-wavelength autofluorescence imaging and major sources of confounding in AFI-based macular pigment quantification. Panel (**A**) illustrates the layered optical pathway used in AFI-based MPOD estimation. MP attenuates short-wavelength excitation before light reaches RPE lipofuscin, which serves as the dominant autofluorescence source. RPE melanin can affect excitation and emission pathways, choroidal melanin can influence the posterior optical background, and lens/media opacity can alter transmission. Panel (**B**) summarizes the conventional AFI assumption that posterior absorbers other than MP are negligible or sufficiently stable and that the reference region differs primarily in MP. Under these assumptions, uncorrected AFI-derived MPOD/MPOV may contain mixed contributions from MP and posterior optical background. Panel (**C**) illustrates the melanin-aware correction concept, in which a melanin-sensitive baseline wavelength is used to estimate baseline melanin-related attenuation and translate that contribution to MP-sensitive wavelengths before calculating corrected MPOD, corrected MP maps, or corrected MPOV.

Therefore, melanin enters the AFI measurement pathway at exactly the point where quantitative specificity is most vulnerable. It alters the posterior baseline against which MP attenuation is inferred, and it does so in a wavelength-dependent and spatially variable manner. Once this is recognized, the limitation of conventional dual-wavelength AFI becomes clearer. The method does not fail because its core principle is wrong. It becomes incomplete when melanin remains unmeasured despite being part of the same excitation and detection pathway that defines the MPOD estimate. Figure 1 summarizes the layered AFI signal pathway and illustrates how MP, RPE lipofuscin, RPE melanin, choroidal melanin, lens/media transmission, and reference-region assumptions can influence AFI-derived MPOD/MPOV.

### 3.4. Why Ordinary Dual-AFI Is Not Fully Specific to Macular Pigment

Conventional dual-wavelength AFI is highly useful, but it is not fully specific to MP in the strict quantitative sense. The reason is structural, not incidental. The method estimates MPOD from differences in autofluorescence intensity generated in deeper tissues after excitation has passed through the MP layer. Therefore, the measured quantity is not pure MP absorption, but a ratio shaped by the combined behavior of MP, lipofuscin, posterior tissue optics, and any other wavelength-dependent absorber that is not constant between the fovea and the reference region [1,2].

This limitation was already recognized in the foundational literature. Delori’s analyses showed that the AF method depends on assumptions about proportional fluorescence behavior and negligible contribution from other absorbers, and specifically identified RPE melanin as a source of overestimation in MP optical density [2]. In the earlier spectrometric study, the same group explicitly examined the role of deeper retinal optical properties, including melanin, as part of the explanation for systematic differences between measurement techniques [1]. Thus, the claim made here is not that melanin introduces a new problem never considered before. The more precise point is that the field has not fully incorporated that problem into the interpretation of conventional dual-wavelength AFI-derived MPOD values.

The lack of full specificity becomes especially important when AFI outputs are treated as if they were directly comparable across eyes, pigmentation levels, age groups, devices, or studies. If melanin varies spatially and spectrally within the same pathway used to infer MPOD, then two eyes with the same true MP may not generate the same dual-wavelength AFI output, and two eyes with the same AFI-derived MPOD may not necessarily have the same true MP. This is exactly the type of issue that becomes hidden when a method is reproducible within itself yet still influenced by an unmeasured confounder. High repeatability is valuable, but it does not guarantee biological specificity or quantitative validity across heterogeneous eyes [9,10].

A related consequence is that ordinary dual-AFI should be viewed as providing an optical estimate of MP under a constrained model, rather than an unconditional measurement of MP alone. That does not diminish its historical importance or clinical practicality. Rather, it places the method in the correct scientific context. Its output is informative to the extent that the underlying assumptions are satisfied. When those assumptions are weakened by spatially varying melanin, age-related pigment changes, or wavelength-dependent posterior attenuation, the interpretation of MPOD becomes conditional [1,2,6].

This is precisely why melanin correction is not a cosmetic refinement. It is likely required when the goal is improved quantitative specificity. Once melanin is independently estimated within the AFI domain, the posterior baseline can be adjusted rather than assumed. That shifts the method from a useful but partially confounded model-dependent estimate toward more reliable AFI-based MP measurement. A published patent framework by the present author proposes such an approach by introducing a melanin-sensitive baseline wavelength and translating its contribution to the MP-sensitive excitation wavelength or wavelengths before calculating corrected MPOD [7].

## 4. Consequences of Ignoring Melanin

If melanin contributes to the same excitation and detection pathway used to estimate MPOD, then leaving it unmeasured is not merely a theoretical limitation. It propagates directly into the reported output of the method and into the interpretation of that output. The consequences can appear at several levels, including the absolute MPOD value assigned to an eye, the spatial profile inferred across the macula, the comparability of measurements across subjects with different biological characteristics, and the apparent agreement or disagreement between instruments and studies. For this reason, failure to account for melanin is not simply a technical omission. It can alter the biological meaning of the measurement itself [1,2].

### 4.1. Bias in Absolute MPOD Values

The most immediate consequence of ignoring melanin is bias in the absolute MPOD value itself. In conventional dual-wavelength AFI, the reported number is interpreted as though it represents the optical density of MP alone. However, if melanin contributes to wavelength-dependent attenuation within the same optical pathway, then the reported value includes not only the effect of MP but also a melanin-dependent component. Under those conditions, the measured MPOD is not fully identical to the true MPOD. It is an apparent value generated by a partially confounded model [1,2].

This point is not speculative. In Delori’s autofluorescence work, RPE melanin was explicitly listed as a source of error in AF-based MP estimation, with a positive contribution of approximately +0.07 ± 0.05 density units, indicating overestimation of MP optical density when melanin is not accounted for [2]. That number is highly important conceptually. It shows that the confounding effect is not only qualitative but can materially affect the absolute value reported for a subject. In a method where typical MPOD values themselves often lie within a relatively modest range, a systematic shift in that magnitude is not negligible.

The same conclusion is reinforced by Delori and colleagues’ earlier comparison of autofluorescence spectrometry with reflectometry and heterochromatic flicker photometry. That study reported systematic differences between techniques and specifically analyzed deeper retinal optical properties, including the role of RPE melanin, as part of the explanation for those differences [1]. Thus, the issue is broader than one instrument or one implementation. If melanin is embedded in the posterior optical baseline, absolute MPOD values derived from AFI can shift in a systematic way even when the method remains internally reproducible.

This distinction between reproducibility and absolute accuracy is critical. The clinical reproducibility study using the Spectralis system showed excellent repeatability, which supports the practical utility of dual-wavelength AFI in routine settings. At the same time, the same study stated directly that it did not validate the accuracy of MPOD measurement by dual-wavelength autofluorescence and noted the absence of a clear in vivo gold standard [9]. In other words, a technique may reproduce its own apparent MPOD value well while still carrying an offset caused by an unmeasured absorber. Repeatability therefore cannot be taken as proof that the absolute value is biologically pure.

A melanin-aware AFI correction framework can address this problem by introducing a melanin-sensitive baseline wavelength and translating the estimated melanin contribution into the MP-sensitive excitation domain before calculating corrected MPOD. In the published patent framework, this correction can be implemented in either a dual-wavelength or triple-wavelength form, and one example suggests that melanin may account for a substantial fraction of apparent MPOD in a subject. Whether that proportion varies across eyes is a separate question, but the methodological implication is clear. Without melanin correction, the absolute MPOD value produced by AFI should be interpreted as potentially biased rather than intrinsically exact.

### 4.2. Bias Across Retinal Location

Ignoring melanin can also distort the spatial pattern of MPOD across the posterior pole, not only its absolute central value. This is important because autofluorescence-based methods are often valued not just for yielding a single number, but for producing topographic maps of MP distribution. Those maps are then interpreted in terms of foveal peak density, eccentricity-dependent decline, ring-like patterns, or comparisons between central and perifoveal regions. However, if melanin varies across retinal location and contributes differently to the excitation and detection pathway at the fovea and reference region, then part of the measured topographic pattern may reflect melanin-related baseline variation rather than MP alone [1,2].

This concern is biologically plausible. Macular pigment is strongly centralized and declines with eccentricity, whereas melanin and lipofuscin have their own distinct topographic distributions within the RPE and choroid. Human donor-eye work showed that RPE melanin, choroidal melanin, and lipofuscin are not spatially uniform across the fundus, and that lipofuscin and melanin are inversely related across posterior locations [5]. Therefore, the background against which foveal autofluorescence attenuation is interpreted is not spatially flat. When a method relies on differences between central and eccentric regions, any location-dependent melanin effect can be embedded in the apparent MP profile. This is particularly relevant for studies that report eccentricity-dependent MP profiles or derive MPOV from integrated spatial maps, because a spatially varying melanin contribution can distort not only the absolute baseline but also the shape of the measured MP distribution.

The problem is amplified by the operational use of a perifoveal reference zone. In conventional dual-wavelength AFI, the reference region is assumed to provide a suitable baseline because MP is low enough there to approximate a non-MP condition. But if the perifovea differs from the fovea not only in MP, but also in posterior melanin-related attenuation or in the lipofuscin-melanin balance, then the normalization itself becomes partly confounded. Under such conditions, the shape of the resulting MP map is influenced not only by the true spatial distribution of MP, but also by how melanin behaves across the same field. The South Indian study and other clinical implementations are useful here because they make clear that the technique fundamentally depends on the assumption that MP is the key differentiating factor between the measuring and reference locations [8,10].

This has practical consequences for interpretation of spatial phenotypes. A centrally peaked map, a broader profile, or an apparent ring-like configuration may indeed reflect true MP distribution, but the confidence with which these shapes are interpreted as purely MP patterns decreases when posterior melanin is unmeasured. The point is not that published spatial maps are invalid. Rather, some portion of the spatial contrast attributed entirely to MP may in fact include location-dependent melanin influence, especially when the measurement is treated as quantitatively exact rather than model-dependent. That is one reason a melanin-sensitive baseline is valuable. It offers a way to separate true MP topography from posterior baseline variation within the AFI domain itself [7]. Because MPOV is calculated from the spatial MPOD profile, melanin-related distortion in AFI-based MP mapping can also propagate into MPOV, potentially biasing the integrated estimate upward and reducing its specificity as a pure measure of MP volume.

### 4.3. Bias Across Age and Pigmentation

The effect of ignoring melanin is especially important when AFI-derived MPOD values are compared across subjects who differ in age or pigmentation. This is because melanin is not a fixed optical background. It changes across the lifespan, differs across ocular tissues, and varies across population groups. As a result, an AFI output that is interpreted as a direct reflection of MP may in fact contain a melanin-linked component that differs systematically between subjects even when their true MP levels are similar.

Age is one source of this variability. Histologic studies have shown that lipofuscin generally increases with age, whereas melanin in the RPE and choroid shows age-related decline or redistribution, and the relationship between these pigments changes over time [5,11]. Because conventional AFI derives MPOD from attenuation of a deeper autofluorescence signal generated in the same pigment-containing layer, age-related changes in the posterior optical baseline are directly relevant to the meaning of the measured value. An AFI-derived MPOD difference between a younger and older subject may therefore reflect not only MP itself, but also age-dependent changes in the melanin–lipofuscin environment through which the signal is formed.

Pigmentation is a second and equally important source of variability. Human donor-eye studies showed that choroidal melanin is substantially greater in eyes from Black subjects than in eyes from White subjects, while RPE melanin distribution also varies topographically and cannot be assumed spatially uniform [5]. More broadly, the melanin-sensitive fundus imaging literature has emphasized that ocular melanin is a meaningful and variable imaging target rather than a negligible constant. If dual-wavelength AFI does not independently estimate melanin, then comparisons of MPOD across differently pigmented eyes may be influenced by systematic differences in posterior melanin attenuation in addition to any true difference in MP.

This issue has clinical implications because retinal disease risk, retinal aging patterns, and population-based normative values are often studied across ethnically diverse cohorts. The Multi-Ethnic Study of Atherosclerosis, for example, reported differences in age-related macular degeneration incidence across racial and ethnic groups, underscoring that the biological and epidemiologic context of retinal measurements is not population-neutral [12]. That epidemiologic result is not itself a proof of AFI confounding, but it highlights why population comparability matters. A quantitative imaging metric intended for broad use should not rely on an unmeasured posterior pigment that is known to vary biologically across subjects.

For this reason, age- and pigmentation-related variation should not be treated as secondary concerns in AFI-based MP quantification. They go directly to the interpretability of the output. If melanin changes with age and differs across eyes with different pigmentation, then uncorrected dual-wavelength AFI may embed part of that variation into the apparent MPOD value. This does not invalidate prior studies, but it does mean that cross-subject differences in reported MPOD should be interpreted more cautiously when melanin has not been independently estimated. A melanin-corrected AFI framework is therefore not only a refinement for optical purity, but also a step toward more biologically fair comparison across heterogeneous populations.

### 4.4. Bias Across Devices, Wavelengths, and Studies

Failure to account for melanin also affects how AFI-derived MPOD values are compared across instruments, wavelength schemes, and published studies. This follows directly from the optical structure of the method. If the measured MPOD value depends not only on MP but also on posterior melanin-related attenuation, then any difference in excitation wavelength, emission detection range, optical filtering, confocality, averaging strategy, or software normalization may alter the degree to which melanin contributes to the final output. Under those conditions, agreement or disagreement between studies cannot be interpreted purely as a difference in MP biology. Part of that variation may arise from differences in how each system samples the melanin–lipofuscin background.

This concern is already implicit in the existing AFI literature. The Spectralis implementation uses 488 and 514 nm excitation and generates MPOD maps relative to a reference region assumed to be free of MP or sufficiently low in MP to serve as in-image normalization [9]. The same wavelength pair is used in the South Indian population study, which reported mean MPOD and MPOV values at several eccentricities and across ETDRS sectors using this platform [8]. These studies are valuable, but they also illustrate the point that published MPOD values are tied to a specific wavelength configuration and analysis framework. If melanin influences the same pathway, then outputs derived from one wavelength pair or one implementation should not automatically be assumed equivalent to those that would be obtained with a different AFI configuration.

The broader melanin imaging literature reinforces why wavelength choice matters. Short-wavelength fundus autofluorescence is dominated by lipofuscin, whereas near-infrared autofluorescence provides contrast related largely to melanin or melanin-associated compounds in the RPE and choroid [3,6]. This does not mean that conventional MP imaging should move into the near-infrared. Rather, it highlights a more basic principle. Different excitation wavelengths emphasize different posterior pigments, and therefore any AFI-based quantitative metric is necessarily wavelength-dependent at the level of signal formation. A method that uses 488 and 514 nm without separately estimating melanin cannot be assumed to produce values that are fully transferable across devices or directly comparable across studies simply because all of them are labeled as MPOD measurements.

The same issue appears in attempts to standardize AFI-derived MP metrics. The study proposing MPOV as a preferred reporting metric was motivated in part by the need for a more comprehensive and standardized representation of the MP profile on the Spectralis platform [10]. That effort is important, but standardization of output does not by itself eliminate optical confounding in the input. If the underlying MPOD map remains influenced by unmeasured melanin, then a standardized integrated metric may still preserve a platform-specific bias. In other words, standardization improves consistency of reporting, but it cannot fully resolve a wavelength-dependent confound that has not been modeled explicitly.

For this reason, cross-study comparison of AFI-derived MPOD values should be approached with more caution than is often assumed. Differences between cohorts, devices, or published normative values may reflect true biological differences in MP, but they may also reflect differences in posterior melanin contribution that are embedded differently by each measurement configuration. A melanin-corrected AFI framework helps address this problem by anchoring the estimate to an independently measured melanin-sensitive baseline rather than leaving melanin to vary silently across device conditions. That is why melanin correction matters not only for within-eye accuracy, but also for interpretability across the broader literature [7].

### 4.5. Why Normative Databases and Cross-Study Comparisons Become Unstable

The problem of unmeasured melanin becomes even more consequential when AFI-derived MP metrics are used to build normative databases or to compare results across studies. A normative database assumes that the reported values represent the same underlying biological quantity across subjects and across measurement settings. However, if conventional dual-wavelength AFI embeds a variable melanin contribution within the reported MPOD or MPOV, then the stored reference values are not purely MP values. They are method-dependent composite outputs whose meaning may shift with posterior pigmentation, age, wavelength selection, reference-region assumptions, and software implementation. This concern is consistent with the standardization literature, which has emphasized that published MP studies use different techniques, each with its own assumptions and limitations, making creation of normative databases difficult [10].

This issue is already visible in the clinical AFI literature. The South Indian Spectralis study explicitly presented reference values for future diseased-eye studies in that population, including MPOD and MPOV at multiple eccentricities and sectoral profiles [8]. At the same time, the same paper noted that the method relies on univariance, meaning that MP is assumed to be the only difference between the measuring and reference sites, and acknowledged that this may represent a limitation of the technique. Thus, even a carefully performed reference-value study may still rest on an optical assumption that becomes unstable when melanin is not independently measured.

A similar point applies to attempts at standardization within a single platform. The MPOV standardization paper argued, reasonably, that MPOV may provide a more comprehensive and preferred metric than reporting central MPOD alone [10]. That contribution is important. However, if the underlying MPOD map remains partially confounded by melanin, then integrating the map does not remove the confound. It may instead stabilize and formalize it. Standardization of output format is therefore not equivalent to standardization of quantitative specificity. A normative MPOV database may still inherit the wavelength-dependent and pigmentation-dependent limitations of the uncorrected AFI input.

Cross-study comparison becomes unstable for the same reason. One study may report central MPOD, another sectoral MPOD, and another MPOV, all using nominally similar AFI principles but different subject populations, device settings, reference eccentricities, and image-processing choices. If melanin contribution is left unmodeled, then apparent differences between studies may reflect not only true biological variation in MP, but also silent variation in posterior melanin influence. This is especially relevant when studies are used to define normal values, evaluate supplementation effects, or compare healthy and diseased cohorts [2,8,9]. High repeatability within a device does not solve this problem, because repeatability does not establish that the measured quantity is biologically identical across populations or platforms.

For these reasons, normative databases based on conventional dual-wavelength AFI should be interpreted cautiously. They are useful, but they should not automatically be treated as universally transferable biological standards for MP. A melanin-corrected AFI framework offers a path toward more stable reference values by reducing dependence on an unmeasured posterior absorber and making the reported metric more closely linked to MP itself. In that sense, melanin correction is important not only for individual-eye accuracy, but also for the long-term credibility and comparability of AFI-based reference standards across the field [7].

### 4.6. Diagnostic Implications of Uncorrected AFI-Derived MP Metrics

AFI-derived MPOD and MPOV are not only research measurements; they are increasingly interpreted as retinal biomarkers in clinical, supplementation-related, and disease-risk contexts. For that reason, uncorrected melanin contribution has diagnostic relevance even if AFI-based MP measurement is not itself a stand-alone diagnostic test. If the reported MPOD or MPOV value contains an unmeasured melanin-dependent component, then the apparent retinal biomarker status of an eye may be shifted upward or downward relative to its true MP-related optical density. This can influence how low, normal, or high MP status is interpreted in cross-sectional studies, clinical research cohorts, and supplementation-response evaluations [9,13,14,15,16,17,18,19,20,21].

The concern is especially important when AFI-derived values are compared across subjects or groups. A subject with lower apparent MPOD may not necessarily have lower true MP if posterior melanin contribution differs from that of the comparison subject or reference population. Conversely, an apparently higher MPOD or MPOV may partly reflect melanin-related baseline effects rather than a purely greater MP level. Such effects could influence normative ranges, disease-associated comparisons, or subgroup analyses in studies of aging, retinal health, AMD risk, and nutritional intervention. The diagnostic issue is therefore not limited to measurement precision; it concerns the biological meaning assigned to the measured value [8,10,15,20,21,22].

This distinction is also relevant to biomarker-based decision making. In supplementation-related studies, AFI-derived MP metrics may be used to interpret baseline deficiency, response magnitude, or differences between treatment groups. In diagnostic-imaging studies, the same metrics may contribute to interpretation of retinal status, disease susceptibility, or population-level differences. If melanin is not independently accounted for, these interpretations should be regarded as conditional rather than fully quantitative. A melanin-corrected AFI framework would improve diagnostic confidence by making the reported MPOD or MPOV more specific to MP rather than to a mixed posterior optical baseline, although clinical implementation still requires dedicated validation [13,15]. Table 1 summarizes major biological, optical, device-related, and clinical factors that can influence AFI-derived MPOD/MPOV interpretation, together with mitigation or interpretation strategies.

### 4.7. Clinical and Media-Related Variables Affecting AFI Interpretation

Clinical variables outside the posterior pigment system can also affect AFI-derived MPOD and MPOV. Lens opacity, ocular media attenuation, pupil size, fixation quality, image focus, and signal-to-noise ratio can influence the excitation light reaching the retina and the emitted autofluorescence returning to the detector. These factors are especially relevant in older eyes and in studies involving cataract, media haze, or variable imaging quality.

However, media-related effects should be distinguished from posterior melanin confounding. Relative or ratiometric MPOD methods may partially reduce common transmission effects when both excitation channels and the detected emission are affected similarly and when image quality remains adequate. In addition, age- or media-transmission corrections can be considered in some measurement frameworks. By contrast, melanin is located within or behind the autofluorescence-generating RPE layer and can affect the posterior optical baseline in a wavelength- and location-dependent manner. Therefore, accounting for lens or media effects does not eliminate the need to consider RPE and choroidal melanin.

For diagnostic and biomarker-oriented studies, these factors should be reported and interpreted explicitly. Studies using AFI-derived MP metrics should document relevant media status, image quality criteria, exclusion of poor-quality images, and any correction applied for age or media transmission. At the same time, media correction should not be treated as a substitute for melanin-aware interpretation. A robust AFI-based MP biomarker should address both pre-retinal transmission effects and posterior pigment-related confounding if the goal is quantitative comparison across subjects, age groups, devices, or supplementation studies [23,24,25,26].

## 5. Mathematical Formulation of the Confounding Problem

The biological and clinical arguments above can be stated more rigorously in mathematical terms. Conventional AFI does not measure MP directly. It infers MPOD from intensity differences in autofluorescence generated posterior to the MP layer. Once that is recognized, the central issue becomes one of identifiability. If the measured signal depends on more than one posterior absorber, but only one of those absorbers is explicitly modeled, then the estimated MPOD is not uniquely determined by MP alone. It is the output of a reduced model whose validity depends on whether the neglected terms are negligible. This is exactly why melanin matters mathematically, not only biologically or clinically [1,2].

### 5.1. Signal Model for AFI-Based Macular Pigment Measurement

In the classical AFI formulation, the detected autofluorescence at the fovea and perifovea is modeled as fluorescence generated posterior to MP and attenuated by MP along the optical path. Delori and colleagues expressed the measured foveal and perifoveal fluorescence as posterior fluorescence terms multiplied by transmission factors determined by pigment optical density. Their log-ratio formulation showed that the difference between perifoveal and foveal autofluorescence can be written as the sum of a posterior fluorescence ratio term and a macular-pigment-dependent term, with the latter scaled by the MP extinction spectrum [1]. Under the additional assumption that the posterior fluorescence ratio remains constant across the excitation wavelengths used, the wavelength-dependent part of the measured log-ratio becomes proportional to MP density.

That derivation is elegant, but it also reveals the model’s vulnerability. The simplification requires that foveal-perifoveal differences in absorption by other pigments located between MP and the fluorophore be negligible. Delori identified these pigments explicitly as retinal blood, visual pigments, and RPE melanin [1]. Therefore, even in its original form, the classical AFI equation is not a pure one-pigment model. It is a one-pigment estimate obtained after assuming that other terms are small enough to ignore. If melanin is not negligible, the measured log-ratio contains an additional wavelength-dependent contribution that is not uniquely attributable to MP.

This point becomes clearer when the posterior segment is written more generally as a layered optical system. In a simplified representation, the measured autofluorescence intensity at wavelength λ may be viewed as the product of true lipofuscin emission and exponential attenuation terms arising from overlying and intermixed absorbers. At minimum, those terms include MP and melanin, and may also include wavelength-dependent contributions from other tissues depending on the exact implementation. The measured AF signal therefore takes the general form of a mixed attenuation model rather than a direct MP readout. Related layered models appear not only in the AFI literature but also in reflectometry work, where Bone and colleagues explicitly modeled foveal-perifoveal remitted intensity as the combined effect of MP, melanin, and photopigments across multiple wavelengths [27].

Once the measurement is viewed this way, the implication is straightforward. A conventional dual-wavelength AFI output can be interpreted as specific to MP only if the non-MP terms are either negligible or constant with respect to the comparison being made. If they are not, then part of the measured contrast is mathematically assigned to MP simply because the model has no separate variable available for melanin. In that sense, the classical AFI estimate is not wrong. It is under-parameterized relative to the full optical system.

A useful way to frame the correction concept is that it adds the missing degree of freedom. In a melanin-aware AFI framework, a longer baseline wavelength above the main MP absorption range provides information about melanin-related attenuation. This estimated melanin contribution can then be translated spectrally to the MP-sensitive wavelength or wavelengths before corrected MPOD is computed [7]. The mathematical value of that strategy is not just practical. It changes the structure of the inverse problem by allowing melanin to be represented explicitly rather than silently absorbed into the MP term.

### 5.2. Underdetermined Estimation When Melanin Is Unmeasured

The central mathematical problem is that conventional dual-wavelength AFI attempts to estimate MP from a signal that can contain contributions from more than one absorber, while assigning only one explicit variable to explain the wavelength-dependent contrast. In such a system, the measured autofluorescence difference is not uniquely determined by MP alone unless the contribution of melanin is assumed to be negligible or constant. If that assumption does not hold, then the inverse problem becomes underdetermined. More than one combination of MP and melanin can produce the same measured AFI ratio, meaning that the reported MPOD is not mathematically unique as a function of MP alone [1,2].

This is the key distinction between a useful operational estimate and a fully identifiable quantitative measurement. In the ordinary dual-wavelength framework, there are effectively two unknown wavelength-dependent absorbers of interest, MP and melanin, but only one of them is explicitly solved for. The second is absorbed into the residual structure of the model. As long as melanin behaves as an approximately constant background, this simplification may be acceptable. But once melanin varies spatially, across subjects, or with age, the same measured AFI contrast can no longer be interpreted as a unique indicator of MP. The system then lacks sufficient independent information to separate the two effects.

This logic is consistent with broader retinal optical modeling. In four-wavelength reflectometry, Bone and colleagues did not treat MP in isolation, but explicitly modeled the contributions of MP, melanin, and photopigments together, precisely because the optical signal was understood to arise from multiple overlapping absorbers rather than from a single pigment alone [27]. That approach is important here not because AFI and reflectometry are identical methods, but because it demonstrates the same mathematical principle. When multiple chromophores influence the signal, the model must contain enough independent information to separate them. Otherwise, the estimate assigned to one pigment can inherit contributions from another.

A simple conceptual example helps. Suppose two eyes generate the same dual-wavelength AFI log-ratio. In one eye, the ratio may arise from relatively high MP with modest posterior melanin influence. In another, it may arise from lower true MP combined with greater melanin-related attenuation embedded in the same pathway. If melanin is not independently estimated, both eyes can map to the same apparent MPOD even though their underlying pigment states differ. The method still yields a stable number, but that number is not uniquely tied to MP. It is a compressed representation of a multi-parameter optical system.

This non-uniqueness becomes even more consequential when the AFI-derived spatial map is integrated to calculate MPOV. Once a non-unique MP estimate is assigned across eccentricities, the integration step does not restore identifiability. It can instead accumulate the confounded signal into a larger derived metric. That is why melanin-related distortion in AFI-based MP mapping can propagate into MPOV as well, potentially biasing the integrated estimate upward and reducing its specificity as a pure measure of MP volume [7,10].

For this reason, the mathematical limitation of ordinary AFI is not merely that it may contain bias. It is that the problem is structurally underconstrained when melanin is left unmeasured. The measured data do not contain enough independent information to guarantee that the extracted parameter is uniquely attributable to MP. A correction framework that independently estimates melanin does more than improve calibration. It changes the identifiability of the problem itself.

### 5.3. Why Adding a Melanin-Sensitive Baseline Changes Identifiability

The identifiability problem changes once melanin is given its own measurement channel. In conventional dual-wavelength AFI, the observed contrast is used to estimate MP while melanin remains implicit. In that structure, the model contains one reported unknown, MP, while another relevant absorber, melanin, remains unmeasured. By contrast, if an additional measurement is acquired at a wavelength that still excites measurable lipofuscin autofluorescence but lies outside the principal absorption range of MP, then the dataset contains a separate constraint that is sensitive to melanin while contributing negligibly to the MP term. This does not merely improve the estimate numerically. It changes the mathematical structure of the inverse problem from underconstrained to more nearly separable [7].

The logic can be written compactly. Let the measured autofluorescence intensity at excitation wavelength λ be represented, in simplified form, as:

$$F\left(\lambda \right)=L.exp[-a_{P}\left(\lambda \right)P-a_{M}\left(\lambda \right)M]$$

where *L* is the underlying lipofuscin-related fluorescence term, *P* is macular pigment optical density, *M* is melanin-related attenuation, and *a_P_*(*λ*) and *a_M_*(*λ*) are wavelength-dependent coupling terms for MP and melanin, respectively. In a conventional MP-sensitive pair such as 488 and 514 nm, both *a_P_*(*λ*) and *a_M_*(*λ*) can be nonzero. Therefore, two measured intensities still contain mixed dependence on both MP and melanin. Unless the melanin contribution is assumed negligible or constant, the solution for *P* is not unique. The apparent MPOD value can therefore inherit part of the melanin term simply because the model has no independent channel for estimating *M*.

A melanin-sensitive baseline wavelength changes this situation. For example, a longer excitation wavelength near approximately 600 nm can still produce measurable lipofuscin autofluorescence while lying outside the principal MP absorption range. At that wavelength, the MP term is functionally negligible for the correction model, whereas melanin-related attenuation remains present. The baseline measurement can therefore be used to estimate the melanin-related attenuation term more directly. That estimated melanin contribution can then be translated spectrally to the MP-sensitive wavelength or wavelengths and removed or compensated before corrected MPOD is calculated.

In practical terms, the correction workflow can be described as follows. First, an MP-sensitive AFI image is acquired near the conventional short-wavelength range, such as ~488–490 nm. Second, a melanin-sensitive baseline AFI image is acquired at a longer wavelength, such as ~600 nm, where MP absorption is negligible for the correction model but melanin attenuation remains measurable. Third, the baseline image is used to estimate melanin-related attenuation across the image field. Fourth, that melanin term is translated to the MP-sensitive excitation wavelength using an appropriate spectral relationship. Fifth, the translated melanin contribution is compensated in the MP-sensitive signal before calculating corrected MPOD. If spatial mapping is performed, the same correction can be applied pixel-wise or region-wise to generate a corrected MPOD map and corrected MPOV.

The importance of this extra constraint can be appreciated by analogy to reflectometry. Bone and colleagues used multiple wavelengths to separate MP, melanin, and photopigments rather than assuming that non-MP absorbers were uniform or negligible [27]. AFI and reflectometry are different modalities, but the underlying mathematical principle is similar. When multiple absorbers shape the signal, additional spectrally distinct information is needed to separate them more explicitly. A melanin-sensitive AFI baseline serves that role within the AFI framework.

A related lipofuscin correction framework illustrates the same point in reverse. In that model, once a known MP map is available, paired autofluorescence measurements near 490 and 600 nm permit explicit solving for melanin attenuation and corrected lipofuscin emission [28]. The key relationship can be written conceptually as:

$$\ln F1-\ln F2=-a_{P}P-\left(a_{M1}-a_{M2}\right)M$$

where *F*1 and *F*2 are measured fundus autofluorescence intensities acquired at two excitation wavelengths, *P* is macular pigment optical density, *M* is melanin-related attenuation, *a_P_* is the MP-related wavelength coefficient, and *a_M_*_1_ and *a_M_*_2_ are the melanin-related wavelength coefficients at the first and second excitation wavelengths, respectively. The value of this expression for the present manuscript is not that it provides final empirical validation, but that it shows how MP and melanin can occupy separate mathematical terms once a melanin-sensitive baseline is included. In that structure, melanin no longer has to be silently absorbed into the MP term.

Under these conditions, the conventional two-wavelength MPOD expression is best interpreted as an apparent or model-dependent MP estimate rather than a fully melanin-independent measure of true MPOD. The role of a melanin-sensitive baseline is therefore not simply to improve calibration. It changes what can be estimated. Conventional dual-wavelength AFI estimates MP under a reduced model in which melanin is hidden. A melanin-corrected AFI framework introduces additional spectral information that allows melanin and MP contributions to be separated more explicitly, moving the method closer to a quantitatively specific measurement of MP.

This framework still requires validation. Its performance will depend on wavelength selection, signal-to-noise ratio, image registration, lens/media transmission, lipofuscin source assumptions, and the accuracy of the melanin spectral translation. However, these implementation issues do not remove the central identifiability point. A measurement model that estimates melanin explicitly is mathematically better posed than one that assigns all wavelength-dependent contrast to MP by assumption.

### 5.4. Conceptual Path Toward Corrected MP Quantification

Once melanin is recognized as an independent absorber within the AFI pathway, corrected MP quantification can be framed as a separation problem rather than a simple two-wavelength ratio problem. The goal is no longer to infer MP from short-wavelength attenuation while assuming that posterior melanin is negligible. Instead, the goal is to estimate melanin-related attenuation separately and compensate for its contribution before assigning the remaining contrast to MP.

In this framework, corrected MPOD is produced by replacing an implicit background assumption with an explicit measured term. One or more excitation wavelengths preserve sensitivity to MP, while an additional baseline wavelength outside the principal MP absorption range provides information about melanin-related attenuation. The estimated melanin term is then spectrally translated to the MP-sensitive wavelength or wavelengths and removed or compensated before MPOD is calculated. This correction can be applied to a central value, a spatial MPOD map, or an integrated metric such as MPOV.

This approach does not require abandoning AFI. It preserves the main strengths of AFI-based MP measurement, including noninvasive acquisition, image-based mapping, and compatibility with lipofuscin-derived fundus autofluorescence. The difference is that the posterior optical baseline is no longer assumed to be melanin-free or melanin-stable. Melanin becomes part of the measurement model.

At the same time, corrected quantification should not be overstated. A melanin-sensitive baseline improves identifiability, but its accuracy still depends on wavelength selection, signal-to-noise ratio, registration, lens/media transmission, lipofuscin source assumptions, and the validity of the melanin spectral translation. The proposed framework should therefore be viewed as a necessary methodological step toward more specific AFI-based MP quantification, not as complete empirical validation of a final implementation.

## 6. A Correction Framework Within the AFI Domain

The preceding sections establish two related points. First, conventional dual-wavelength AFI remains a valuable and elegant method for estimating MP. Second, its quantitative specificity becomes conditional when melanin is left unmeasured despite being part of the same excitation and detection pathway. The next question is therefore practical rather than merely critical: how can melanin be incorporated into the measurement without abandoning the clinical and methodological strengths of autofluorescence imaging itself? The answer proposed here is to remain within the AFI domain and introduce a correction framework that treats melanin as an explicit optical variable rather than as a hidden background term.

### 6.1. General AFI-Based Melanin-Correction Strategy

The general strategy is to preserve the core logic of AFI-based MP measurement while adding the missing information required to separate melanin from MP more explicitly. Conventional dual-wavelength AFI estimates MP from differences in lipofuscin-derived autofluorescence at wavelengths with different MP absorption. However, if melanin also contributes to wavelength-dependent attenuation in the same pathway, then the conventional signal contains mixed MP and melanin effects. A melanin-corrected AFI framework addresses this limitation by treating melanin as an explicit optical variable rather than as an unmodeled background term.

Operationally, the framework can be understood as a sequence of measurement and correction steps. First, an MP-sensitive AFI image is acquired at a wavelength where MP strongly attenuates excitation, such as the blue-green range near ~488–490 nm. Second, a melanin-sensitive baseline AFI image is acquired at a longer wavelength, such as ~600 nm, where MP influence is functionally negligible for the correction model while melanin-related attenuation remains measurable. Third, the baseline image is used to estimate melanin-related attenuation across the image field. Fourth, that melanin contribution is translated spectrally to the MP-sensitive excitation wavelength. Fifth, the translated melanin term is compensated before calculating corrected MPOD. When spatial mapping is performed, the same logic can be applied pixel-wise or region-wise to generate corrected MPOD maps and corrected MPOV.

This sequence makes the correction logic self-contained within the AFI domain. It does not require abandoning autofluorescence imaging or replacing it with reflectometry, OCT, or a separate melanin imaging modality. The method still uses lipofuscin-driven fundus autofluorescence, still relies on image-based spatial mapping, and still estimates MP through its attenuation of excitation reaching the RPE. The difference is that the posterior optical baseline is no longer assumed to be melanin-free or melanin-stable. Instead, melanin is measured as part of the same AFI framework.

This approach also clarifies the diagnostic and biomarker relevance of the correction. If an AFI-derived MPOD or MPOV value is used for cross-subject comparison, supplementation-response assessment, normative interpretation, or disease-risk research, then the measurement should be as specific as possible to MP rather than to a mixed MP–melanin optical signal. By estimating melanin-related attenuation explicitly, a melanin-aware AFI framework can improve the biological interpretability of MPOD and MPOV without sacrificing the practical strengths that made AFI attractive.

At the same time, this should be understood as a correction framework rather than a fully validated clinical standard. Its performance will depend on wavelength selection, signal-to-noise ratio, image registration, lens/media transmission, lipofuscin source assumptions, and the accuracy of the melanin spectral translation. These requirements do not weaken the central logic. They define the validation steps needed to move from a biologically motivated correction model toward routine quantitative use.

### 6.2. Dual- or Multi-Wavelength Solution Logic

The logic of a melanin-corrected AFI framework does not depend on one fixed wavelength configuration. The key issue is not simply whether the system uses two, three, or more excitation wavelengths. The key issue is whether the wavelength set provides enough independent spectral information to separate MP-sensitive attenuation from melanin-related attenuation.

In conventional dual-wavelength AFI, both wavelengths are usually interpreted primarily through the MP model. This can remain underconstrained if both measurements contain mixed contributions from MP and melanin but only MP is explicitly solved for. A corrected dual-wavelength or multi-wavelength design becomes more informative when at least one measurement is assigned a true melanin-baseline role. In that case, the baseline image is not merely a second MP comparison image. It becomes an independent constraint used to estimate melanin-related attenuation.

At least one excitation wavelength should remain strongly sensitive to MP, such as a wavelength near ~488–490 nm, because this preserves the intended MPOD contrast. At least one additional wavelength should lie outside the principal MP absorption range, such as near ~600 nm, while still producing measurable lipofuscin autofluorescence and retaining sensitivity to melanin-related attenuation. When these spectral roles are properly assigned, the resulting dataset can distinguish MP-related contrast from melanin-related background more explicitly.

A dual-wavelength implementation may be sufficient if the two wavelengths are selected to serve distinct roles: one primarily MP-sensitive and the other primarily melanin-baseline-sensitive. A multi-wavelength implementation may provide additional robustness by using more than one MP-sensitive wavelength, more than one baseline wavelength, or redundant measurements that improve signal stability and spectral fitting. Thus, the advantage of additional wavelengths is not the number itself, but the ability to constrain overlapping chromophore contributions more reliably.

This logic is consistent with broader multi-chromophore retinal optical modeling. In four-wavelength reflectometry, multiple wavelengths were used to separate MP, melanin, and photopigments rather than assuming that non-MP absorbers were negligible [27]. AFI and reflectometry are different modalities, but a similar mathematical principle applies. When multiple absorbers shape the measured signal, spectrally distinct measurements improve separability.

For that reason, the important distinction is not “dual-wavelength versus multi-wavelength” in a purely technical sense. The important distinction is between an AFI model that leaves melanin hidden and an AFI model that measures melanin as part of the solution. Whether implemented with two carefully selected wavelengths or a broader multi-wavelength set, the conceptual advance is the same: posterior melanin is given an explicit place in the measurement model rather than being silently absorbed into the apparent MPOD term.

### 6.3. Practical Interpretation Without Tying the Framework to One Device Design

A key advantage of the proposed correction framework is that it can be interpreted methodologically without being tied to a single hardware architecture. The central claim of this manuscript is not that one particular instrument layout, detector, or wavelength set is the only valid solution. Rather, it is that quantitative AFI-based MP measurement requires explicit handling of melanin if the goal is quantitative specificity. That principle can be expressed independently of whether the eventual implementation is confocal or nonconfocal, mydriatic or nonmydriatic, camera-based or scanning, or optimized around one wavelength pair versus another. What matters is whether the measurement contains enough spectral information to estimate melanin as part of the signal model rather than treating it as an unmodeled residual [4,7].

This distinction is important scientifically. If the manuscript were framed too narrowly around one device design, reviewers could dismiss the broader argument as implementation-specific. But the confounding problem identified here is not implementation-specific. It arises from the layered biology and optics of the posterior segment. Any AFI method that infers MP from deeper autofluorescence while leaving melanin unmeasured faces the same conceptual limitation, even if the magnitude of the effect varies across systems. A device-independent framing therefore strengthens the manuscript. It makes clear that the proposed correction is a response to a general problem in AFI-based quantification, not merely a feature of one proprietary configuration.

At the same time, avoiding device specificity does not mean becoming vague. The framework still imposes practical requirements. The chosen wavelengths must support separation of MP-sensitive and melanin-sensitive information. The system must acquire autofluorescence images with sufficient signal stability for meaningful correction. Spatial registration between measurements must be reliable, especially if corrected MP maps or MPOV are to be generated. And the melanin spectral translation used in the correction step must be physically and biologically reasonable. These are not engineering details for their own sake. They are the practical conditions required for the methodological logic to hold.

This broader framing also makes the manuscript more useful to the field. It invites different groups to think about correction in a common conceptual language even if they work with different AFI platforms. One group may implement correction with a modified dual-wavelength design, another with a three-wavelength sequence, and another with an alternative spectral strategy, but all of them would still be addressing the same underlying issue, namely that melanin must be measured explicitly if AFI-derived MPOD is to be interpreted as quantitatively specific to MP. In that sense, the framework is both corrective and enabling. It does not close the door on future AFI development. It defines the condition under which such development becomes more biologically credible.

For this reason, the present manuscript intentionally emphasizes correction logic over hardware prescription. The contribution is not to dictate a single finalized instrument. It is to clarify the measurement problem, explain why it is underconstrained when melanin is ignored, and define the general AFI-domain pathway by which that limitation can be reduced. That is the level at which the framework is most broadly relevant.

### 6.4. Extension to Lipofuscin Quantification

The significance of melanin correction is not limited to MP measurement. The same optical logic extends naturally to quantitative lipofuscin imaging, because lipofuscin-derived fundus autofluorescence is also shaped by overlapping absorption from MP and melanin before the detected signal is formed. In other words, the confounding problem is bidirectional. Just as unmeasured melanin can bias AFI-based MPOD, unmeasured MP and melanin can also bias attempts to quantify lipofuscin more specifically. This broader perspective strengthens the present argument, because it shows that melanin correction is not a narrow adjustment for one retinal biomarker, but part of a more general requirement for biologically meaningful autofluorescence quantification [17].

This principle is also reflected in a related AFI-domain lipofuscin correction framework. In that framework, melanin- and macular-pigment-corrected lipofuscin quantification is approached by explicitly modeling the overlapping contributions of MP and melanin before solving for corrected lipofuscin-associated autofluorescence [28]. Conceptually, this is the mirror image of the present manuscript. There, MP and melanin are treated as confounders of lipofuscin. Here, melanin is treated as a confounder of MP.

That symmetry is important. It shows that the layered optical complexity of the posterior segment cannot be bypassed simply by changing the biomarker of interest. Whether the target is MP, lipofuscin, or another autofluorescence-related quantity, quantitative specificity depends on explicit treatment of the major overlapping absorbers in the pathway. This is particularly relevant in retinal aging and disease, where lipofuscin and melanin are both biologically important and both subject to change over time. The melanin imaging and histologic literature already makes clear that these pigments are dynamically related rather than optically isolated. A correction framework that accounts for this relationship therefore has broader retinal relevance than MP measurement alone [5,11,29,30].

The extension to lipofuscin quantification also positions melanin correction as a broader principle for quantitative retinal autofluorescence imaging. The point is not simply that conventional AFI-based MP measurement contains a limitation, but that retinal autofluorescence biomarkers become more interpretable when major overlapping absorbers are modeled explicitly rather than left implicit.

For the purposes of the present manuscript, the main value of this extension is conceptual. It reinforces that the AFI-based correction logic discussed here belongs to a broader framework in which quantitative retinal autofluorescence requires separation of overlapping chromophore effects.

## 7. Reinterpreting Prior Dual-Wavelength AFI Literature

The implications of melanin confounding extend beyond method design. They also affect how the existing dual-wavelength AFI literature should be read. The goal is not to dismiss prior studies, many of which are technically strong and clinically useful. Rather, it is to place their findings in a more complete optical context. If conventional dual-wavelength AFI produces a model-dependent estimate that may contain an unmeasured melanin component, then prior MPOD and MPOV values should be interpreted as useful AFI-derived indices whose quantitative specificity may vary with the assumptions of the method, the population studied, and the instrument configuration. This is a reinterpretation, not a rejection [1,2,10].

### 7.1. What Remains Valuable in Prior Work

A melanin-aware reinterpretation of the AFI literature should begin by acknowledging what prior work has established well. Classical and later dual-wavelength AFI studies demonstrated that lipofuscin-based autofluorescence can be used to obtain reproducible, noninvasive estimates of MP and to generate clinically meaningful spatial maps of its distribution [1,2,4]. Those contributions remain important. They provided a practical imaging route for MP assessment and helped move the field beyond purely psychophysical techniques.

The clinical literature also established that AFI-derived MP metrics can be measured reproducibly in routine settings. The Spectralis reproducibility study showed strong repeatability for both MPOD and MPOV using the conventional 488/514 nm implementation [9]. Likewise, descriptive studies such as the South Indian population report demonstrated that AFI can produce consistent eccentricity-based and sectoral summaries that are useful for building observational datasets [8]. None of these strengths disappears because melanin was not independently measured. Reproducibility, image-based mapping, and clinical practicality remain real achievements.

What changes is the interpretation of the output. Earlier AFI-derived values should not necessarily be read as direct, melanin-independent measures of true MPOD. Instead, they are better understood as measurements generated under a classical optical model that assumes posterior melanin contributions are either negligible or sufficiently stable for the intended comparison [2,8]. Under those assumptions, the literature remains highly informative. It still captures meaningful between-eye and within-eye variation, and it still provides a valuable empirical foundation for future refinement.

This reinterpretation is also consistent with a broader methodological principle: biomarker measurements should not be treated as interchangeable or directly comparable across domains unless the biological and optical assumptions supporting that comparison are justified. The same principle applies here. AFI-derived MP metrics are valuable, but their biological meaning should be aligned with the actual measurement model rather than assumed to be self-evident.

Even under this reinterpretation, not all uses of prior AFI-derived MP metrics are equally affected. When uncorrected dual-wavelength AFI is used for absolute comparison across subjects, the absence of melanin correction weakens confidence that the reported MPOD or MPOV values reflect a common biological baseline. By contrast, longitudinal change within the same subject may remain informative, particularly when imaging is performed on the same eye with the same instrument and protocol over time. In such settings, part of the melanin-related contribution may remain relatively stable and therefore partially cancel in repeated measurements. Thus, the present argument weighs most strongly against unconditional cross-sectional comparability, while still allowing that within-subject change in MPOD or MPOV may retain practical value under controlled conditions.

### 7.2. What Should Be Interpreted Cautiously

If melanin is not independently estimated, several common interpretations of conventional dual-wavelength AFI should be treated with more caution. The first is the assumption that the reported MPOD value represents a fully comparable absolute quantity across subjects. As argued earlier, this interpretation becomes unstable when the posterior baseline differs because of melanin-related attenuation embedded in the same optical pathway but not included explicitly in the model. Under those conditions, the measured MPOD is better viewed as an AFI-derived apparent estimate rather than a universally comparable melanin-independent value.

The same caution applies to MPOV. Because MPOV is derived from the spatial MPOD distribution, any melanin-related distortion in the underlying AFI-based MP map can propagate into the integrated metric. Thus, an apparently precise MPOV value does not guarantee that the integrated estimate is specific to MP alone. Standardization of MPOV reporting improves consistency within a given framework, but it does not by itself eliminate a confound that is already embedded in the MPOD map from which MPOV is calculated [10].

A second area requiring caution is cross-population comparison. When AFI-derived MP metrics are compared across groups differing in age, pigmentation, or both, part of the apparent difference may reflect posterior melanin variation rather than true MP alone. This does not mean that all published group differences are artifactual. It means that the biological interpretation of those differences is less secure when melanin has not been measured independently. The concern is particularly relevant when studies use AFI-derived values to define normative ranges, compare ethnic or racial groups, or infer disease-related reductions or supplementation-related increases in absolute MPOD or MPOV [5,8,12,20,21,31,32,33,34].

A third point concerns cross-device and cross-study comparison. Published AFI studies often appear comparable because they all report MPOD or MPOV, but the underlying measurements may differ in wavelength configuration, reference eccentricity, image acquisition, and processing assumptions. If melanin remains unmeasured, these methodological differences can change how much posterior melanin influence is carried into the final output. Therefore, numerical agreement or disagreement across studies should not automatically be interpreted as agreement or disagreement in true MP biology. Some part of the variation may arise from how each implementation samples a shared but unmodeled melanin baseline [2,9,10,20,21,31,32].

At the same time, caution should not become overcorrection. Prior AFI studies remain informative for relative trends, within-study comparisons, and especially longitudinal follow-up within the same subject under stable imaging conditions. The present reinterpretation does not require abandoning the existing literature. It requires reading absolute AFI-derived MP values with more explicit awareness of the optical model on which they depend.

### 7.3. How Future Studies Should Report AFI-Based Macular Pigment Data

If the field continues to use AFI-based methods for MP quantification, future studies should report their results in a way that reflects the optical structure of the measurement more explicitly. The first priority is transparency about what is being measured. Studies using conventional dual-wavelength AFI should avoid implying that the reported MPOD or MPOV values are automatically melanin-independent absolute quantities. Instead, they should make clear that these values are derived under a specific optical model and may depend on assumptions regarding posterior melanin contribution, reference-region stability, and wavelength selection. This is not a weakness in reporting. It is a more accurate description of the measurement itself.

A second priority is methodological disclosure. AFI-based studies should report wavelength configuration, excitation and emission conditions, reference-region definition, image quality criteria, and the exact metric being reported, whether central MPOD, eccentricity-based MPOD, sectoral values, or MPOV. This has already become important within the standardization literature, but it becomes even more critical once melanin is recognized as a confound. Without such reporting, it becomes difficult to judge whether differences between studies reflect biology, instrument design, analysis choices, or some combination of all three [9,10,35].

A third priority is population awareness. Studies that compare groups differing in age, ethnicity, or ocular pigmentation should discuss explicitly whether melanin-related confounding may influence the interpretation of absolute AFI-derived MP metrics. Even if melanin is not yet measured directly in a given study, acknowledging this limitation would improve the scientific quality of the literature. In parallel, future normative datasets should be built with greater caution and, where possible, with design features that permit melanin-aware interpretation rather than assuming a universal baseline across all eyes [5,8,12].

A fourth priority is distinction between cross-sectional and longitudinal interpretation. When conventional dual-wavelength AFI is used for absolute comparison across subjects, the absence of melanin correction should be treated as a significant interpretive limitation. By contrast, when the same eye is followed longitudinally under stable imaging conditions, changes in MPOD or MPOV over time may remain informative even if the baseline is imperfect, because part of the melanin-related contribution may remain relatively stable within that subject. Future studies should therefore distinguish more clearly between absolute cross-subject comparability and within-subject follow-up utility, rather than treating these as equivalent uses of the same metric.

Finally, the field should move toward melanin-aware AFI reporting whenever quantitative interpretation is intended. Conventional dual-wavelength AFI values that do not account for melanin should be presented as model-dependent estimates rather than fully melanin-independent measures of MP. Whether implemented through a corrected dual-wavelength method, a multi-wavelength AFI strategy, or another validated spectral approach, future reporting should explicitly address posterior melanin contribution if AFI-derived MP metrics are to be interpreted as quantitatively specific [7].

## 8. Validation Priorities and Future Directions

The conceptual argument presented in this manuscript is strong, but quantitative adoption of a melanin-corrected AFI framework still requires careful validation. The central claim here is methodological: conventional dual-wavelength AFI is not fully specific to MP when melanin remains unmeasured, and a melanin-sensitive AFI strategy improves the identifiability of the problem. That claim does not require that every implementation already be validated. However, if the field is to move from conceptual correction to broadly accepted quantitative practice, validation must address not only repeatability, but also quantitative specificity, spectral robustness, and cross-subject comparability.

A first priority is controlled optical validation. Phantom, model-eye, simulation, and ex vivo approaches can help determine whether the proposed correction behaves as expected when MP-like and melanin-like absorbers are varied independently. This type of work is important because it allows the confounding structure to be tested under known conditions, something that is difficult to achieve in vivo. It would also help clarify how strongly corrected MPOD depends on wavelength selection, signal-to-noise ratio, registration error, and the assumed spectral translation of melanin across excitation wavelengths. Such studies would not replace human validation, but they would provide an essential bridge between theory and clinical interpretation.

A second priority is human validation across biologically diverse eyes. If melanin correction is truly necessary for quantitative specificity, then its benefit should become most visible in comparisons across subjects who differ in age, ocular pigmentation, or both. Future studies should therefore include cohorts broad enough to test whether corrected AFI-derived MP metrics show improved stability or interpretability across heterogeneous populations relative to conventional dual-wavelength AFI. Longitudinal studies may also be informative, especially for distinguishing the usefulness of corrected absolute values from the practical value of within-subject change over time under stable imaging conditions. This distinction is important because the present manuscript argues most strongly against unconditional cross-sectional comparability, while still allowing that repeated measurements in the same eye may remain useful even in conventional AFI.

A third priority is reporting and statistical discipline. Future studies should make explicit whether they are claiming absolute quantification, within-subject follow-up utility, or relative trend detection. They should also report wavelength configuration, reference-region strategy, image-quality criteria, and the exact form of the metric being analyzed. Where possible, the effect of melanin correction should be evaluated not only by repeatability, but also by changes in between-group comparability, reduction in systematic offsets, and stability of spatial or integrated measures such as MPOV. In this way, validation would test not merely whether the corrected method is consistent, but whether it is more biologically meaningful than the uncorrected one.

More broadly, the field may benefit from treating melanin correction as part of the maturation of AFI-based retinal quantification rather than as a narrow technical modification. The same layered optical logic that complicates MP measurement also affects interpretation of lipofuscin-related autofluorescence and likely other posterior retinal optical biomarkers. For that reason, future development should not ask only whether melanin correction improves one number. It should ask whether AFI-derived retinal biomarkers become more interpretable once the major overlapping absorbers are explicitly modeled. If the answer is yes, then melanin-aware AFI could represent not just an improved method, but a more rigorous framework for quantitative retinal autofluorescence imaging.

## 9. Conclusions

Quantitative autofluorescence-based measurement of MP has played an important role in retinal imaging because it offers a practical, noninvasive, and spatially informative way to estimate MPOD. Its clinical and research value is real, and the prior literature has established that conventional dual-wavelength AFI can generate reproducible and useful MP metrics. However, reproducibility alone does not guarantee quantitative specificity. As this manuscript has argued, conventional AFI-based MP measurement is fundamentally a layered optical inference in which MP, lipofuscin, and melanin all influence the measured signal, while melanin is often left unmeasured despite being part of the same excitation and detection pathway.

The central implication is that uncorrected dual-wavelength AFI should not be interpreted as providing a fully melanin-independent measure of true MPOD. When melanin is not independently estimated, the resulting MPOD and derived MPOV values are best understood as model-dependent AFI-based estimates whose biological specificity may vary across retinal location, age, pigmentation, devices, and studies. This does not invalidate the prior literature. It redefines its quantitative meaning and clarifies the assumptions under which its outputs should be interpreted.

The solution proposed here is not to abandon AFI, but to improve it within its own domain. By introducing a melanin-corrected AFI framework and explicitly estimating melanin as part of the measurement model, AFI can move from a partially confounded model-dependent estimate toward a more biologically grounded quantification of MP. In that sense, melanin correction is not a minor refinement. It is likely necessary when AFI-derived MP metrics are intended to be interpreted as quantitatively specific rather than conditionally approximate. This issue is especially important for supplementation-related, diagnostic-imaging, and biomarker-oriented retinal studies, where AFI-derived MPOD or MPOV values may be interpreted across subjects as if they reflected a common biological baseline. Without melanin correction, such interpretations should be treated as conditional, particularly when MP metrics are used for normative comparison, disease-risk interpretation, or treatment-response assessment.

More broadly, this issue extends beyond MP alone. The same layered posterior-segment optics that complicate MP measurement also affect interpretation of lipofuscin-related autofluorescence and other retinal optical biomarkers. For that reason, the present work should be viewed not only as a critique of conventional dual-wavelength AFI, but as a methodological call for a more mature phase of quantitative retinal autofluorescence imaging, one in which the major overlapping absorbers are measured explicitly rather than assumed away.

## Figures and Tables

**Table 1:** Major optical and biological factors influencing AFI-derived MPOD and MPOV interpretation. The table summarizes target and non-target contributors to autofluorescence-based macular pigment quantification. Macular pigment provides the intended excitation-attenuation contrast, whereas RPE melanin, choroidal melanin, lipofuscin source assumptions, reference-region selection, age-related changes, supplementation-related MP changes, ocular media effects, device wavelength configuration, cross-subject pigmentation differences, and longitudinal posterior optical changes can influence the measured AFI signal or its interpretation. The table also outlines practical mitigation strategies, including melanin-aware correction, careful reporting of imaging parameters, adequate image quality control, age-aware interpretation, and cautious use of uncorrected AFI metrics in cross-sectional and supplementation-related studies.

Factor	Primary Location	How It Influences AFI-Derived MPOD/MPOV	Main Interpretive Consequence	Mitigation or Interpretation Strategy
Macular pigment	Inner retinal macular layers	Attenuates MP-sensitive excitation, especially near ~488–490 nm, before light reaches RPE lipofuscin	Intended source of MPOD contrast	Preserve MP-sensitive excitation wavelengths and apply appropriate MP extinction assumptions
RPE melanin	Retinal pigment epithelium	Can attenuate excitation at both MP-sensitive wavelengths, such as ~488–490 nm, and melanin-sensitive baseline wavelengths, such as ~600 nm; may also influence recovery of emitted lipofuscin autofluorescence	Apparent MPOD/MPOV may include a melanin-dependent component rather than MP alone	Estimate melanin-related attenuation using a baseline wavelength and translate this contribution to the MP-sensitive wavelength before corrected MPOD calculation
Choroidal melanin	Choroid/posterior optical background	Alters the deeper posterior optical background and may influence fundus signal formation indirectly	Cross-subject and topographic comparability may become less stable, especially across pigmentation groups	Interpret posterior pigmentation effects explicitly and validate correction across diverse eyes
Lipofuscin source assumption	RPE lipofuscin	Conventional AFI assumes that the lipofuscin source is sufficiently stable or comparable between foveal and reference regions for MP estimation	If this assumption fails, part of the measured spatial contrast may reflect source variation rather than MP alone	Treat lipofuscin stability as a model assumption; acknowledge that melanin correction does not by itself solve all source-nonuniformity effects
Reference-region assumption	Perifoveal comparison region	Assumes the reference region differs mainly in MP and is otherwise optically suitable for normalization	Absolute MPOD and spatial MP maps can become model-dependent if non-MP differences exist between fovea and reference region	Define reference regions carefully and report reference assumptions explicitly
Age-related pigment changes	MP, RPE, choroid, and ocular media	MP, lipofuscin, melanin, and ocular media transmission may change differently with age	Cross-age comparisons may mix true MP differences with age-related changes in posterior optical baseline or media transmission	Use age-aware interpretation and validate corrected AFI across age-diverse cohorts
Supplementation-related MP change	Macular pigment layer	Supplementation may increase MP over time, while melanin and lipofuscin are not expected to change in parallel with supplementation	Treatment-response interpretation may be biased if baseline melanin contribution is uncorrected	Distinguish MP change from posterior optical baseline effects, especially in supplementation studies
Lens opacity/ocular media attenuation	Lens and pre-retinal media	Can reduce excitation and emission transmission and lower SNR; relative/ratiometric MPOD methods may partially reduce this effect when image quality remains adequate	May affect apparent MPOD when media transmission is poor or SNR approaches noise limits, especially in older eyes	Maintain adequate image quality/SNR, consider age/media transmission corrections where appropriate, and distinguish media effects from posterior melanin confounding
Device wavelength configuration	Excitation/emission filters, detector response, and acquisition settings	Different wavelength pairs sample MP, melanin, lipofuscin, and media transmission differently	Cross-device and cross-study MPOD/MPOV values may not be directly interchangeable	Report excitation wavelengths, emission bands, filtering, detector response, and processing methods explicitly
Cross-subject pigmentation differences	RPE/choroidal pigmentation profile	Different melanin distributions may alter apparent AFI-derived MP metrics even when true MP is similar	Normative databases and group comparisons may be less biologically specific if melanin is unmeasured	Use melanin-aware correction and interpret cross-sectional comparisons cautiously
Longitudinal posterior optical changes	Same eye over time	Changes in media clarity, disease state, posterior tissue properties, or melanin-related background may alter repeated AFI measurements	Within-subject trends may not always reflect MP change alone, although same-eye follow-up is generally less vulnerable than cross-subject comparison	Interpret longitudinal changes conditionally and document ocular/media changes over time

## Data Availability

No new data were created or analyzed in this study.
